# Editorial: Transport of Nutrients, Metabolites and Ions linked to Bioenergetics: Relevance to Human Pathology, Volume II

**DOI:** 10.3389/fmolb.2022.957599

**Published:** 2022-08-17

**Authors:** Mariafrancesca Scalise, Piotr Koprowski, Cesare Indiveri

**Affiliations:** ^1^ Department DiBEST (Biologia, Ecologia, Scienze della Terra) Unit of Biochemistry and Molecular Biotechnology, University of Calabria, Rende, Italy; ^2^ Nencki Institute of Experimental Biology (PAS), Warsaw, Poland

**Keywords:** membrane transport, pathology, human health, channels, cell biology

The Research Topic on “*Transport of Nutrients, Metabolites, and Ions linked to Bioenergetics: Relevance to Human Pathology*” volume 2, includes 6 contributions among which are 4 original articles, 1 brief research report, and 1 review. The second volume of the Research Topic follows the main scope of the first volume, i.e., adding novel and relevant information on the role of membrane transporters in bioenergetics. In particular, both aspects of basic and applied research are dealt with.

The fundamental role played by membrane transporters in life is unequivocally testified by the occurrence of several cell dysfunctions occurring in all living organisms when these proteins are mutated, de-regulated, misfunctional, and/or mislocalized. Indeed, membrane transporters ensure the maintenance of the homeorhesis, that is the proper flux of metabolites, ions, and waste compounds required for cell homeostasis. Furthermore, during evolution, the membrane transporters acquired also the ability to mediate the uptake and efflux of xenobiotics, including drugs. This feature attracted the attention of pharma companies that nowadays focus a large body of their work on studying membrane transporters as both targets and transporters of novel and repurposed drugs.

Notwithstanding the well-assessed function(s) of these proteins in cell life, several gaps exist in the knowledge of functional, structural, and regulatory properties of membrane transporters. In this frame, the original articles of the Research Topic, volume 2, find a scientific placement. Regarding functional characterization, Albrecht’s group performed a study describing the role of membrane transporters critical for placenta homeostasis. The paper evaluated the expression changes occurring during the first weeks of pregnancy, upon trophoblast differentiation. Very interestingly, the study highlighted that the glucose transporter (SLC2A1), the amino acid transporter (SLC7A5/SLC3A2), the iron transporter (TfR1), and the cholesterol transporters (ABCA1, ABCG1), increase their expression during placenta formation. This finding is in perfect agreement with the increased need for nutrients in the fetus development; indeed, the transporters under investigation being out of balance triggers compromised fetal growth Karahoda et al.

The second article on the Research Topic is from Geyer’s group and deals with the characterization of rare genetic variants of the transporter SLC10A7, a negative regulator of intracellular calcium signaling. Therefore, is not surprising that mutations occurring at the level of the human gene are causative of severe diseases linked with alteration of calcium flux in cells. The study functionally characterized six SLC7A10 variants Wannowius et al. employing combined approaches of site-directed mutagenesis, *in situ* localization, and fluorescent analyses of Ca^2+^ fluxes.

The third original article is from Fredriksson’s group and deals with the regulatory role played by SLC38A10 in glutamate homeostasis. Indeed, glutamate concentration needs to be maintained constant in synaptic clefts for allowing proper neurotransmission. In this scenario, the plasma membrane transporter SLC38A10 is considered a transceptor allowing the regulation of glutamate sensing through mTORC1 signaling. The paper shows data deriving from combined approaches of transcriptomic, protein arrays, and metabolomic. Interestingly, alterations of SLC38A10 are linked with lowering of cholesterol content, involved in neurodegeneration Tripathi et al.

The last original article on the Research Topic is from Qu’s group and sheds light on the structure/function relationship of another group of glutamate transporters, belonging to the SLC1 family. Indeed, this family includes five high-affinity glutamate transporters mainly located in the brain, where they are responsible for glutamate reuptake to reduce excitotoxicity in synaptic clefts. The article, by employing site-directed mutagenesis coupled with chemical targeting, demonstrated that when EAAT2 interacts with substrate, the TM3-TM4 loop undergoes a proximal conformational change to TM7 allowing the transport cycle to occur with the elevator mechanism typical of this transporter family Qu et al.

The brief research report from Weide’s group deals with the identification of the protein Pals1 as responsible for a key process in kidney homeostasis. Indeed, Pals1 is a member of the Crumbs protein complex that regulates cell polarization and junction formation. The transcriptome of Pals1-mice knockout highlighted the strong deregulation of several members of the SLC superfamily. The most affected ones are key players in kidney physiology, i.e., glucose transporter (SLC2 and SLC5), the sodium- and chloride-dependent neurotransmitter transporter family (SLC6 group), the amino acids transporters (SLC7 group), monocarboxylate transporters (SLC16 group), and organic cationic, and zwitterions transporters (SLC22 group). This has been linked with cystic formation typical of several kidney pathological conditions Berghaus et al.

The last article on the Research Topic is a review from Sucic’s group. The review is an interesting and comprehensive state of the art on the GABA transporters from the SLC6 family; indeed, the human genome encodes four isoforms of GABA transporters that are involved in modulating neurotransmission. The key relevant role of these proteins is demonstrated by the occurrence of several neurological disorders with a broad range of severity depending on the type of mutations. Over the years, a sizable number of mutations have been annotated on the genes encoding GABA transporters. The effect of single mutants has been studied using the *Drosophila melanogaster* model which allows relatively fast and reproducible investigations. This is particularly relevant in drug discovery approaches considering that GABA transporters became hot targets for treating epilepsy, schizophrenia, and intellectual disabilities Fischer et al..

As a concluding remark of this volume 2, it can be highlighted that the Research Topic added further important novelties to the very broad and still incomplete field of membrane transporters. Notwithstanding, the full picture is still a low-resolution image, and its improvement represents a long-term challenge that requires substantial efforts in terms of experimental approaches and Research Topic and analysis of data. In this respect, multidisciplinary platforms including in silico, *in vitro*, *in vivo*, and artificial intelligence tools represent a mandatory step forward to make a decisive increase in our knowledge of membrane transport phenomena.

